# The effect of social network based motivational interviewing on health behaviors among infertile women with polycystic ovary syndrome: A randomized controlled trial

**DOI:** 10.1038/s41598-024-61161-9

**Published:** 2024-05-27

**Authors:** Zeinab Hamzehgardeshi, Forouzan Elyasi, Mahmood Moosazadeh, Imaneh Ahmadi, Shayesteh Jahanfar, Maryam Ahmadian, Fatemeh Ansari

**Affiliations:** 1 Sexual and Reproductive Health Research Center, Mazandaran University of Medical Sciences, Sari, Iran; 2 Department of Reproductive Health and Midwifery, Mazandaran University of Medical Sciences, Sari, Iran; 3 Psychiatry and Behavioral Sciences Research Center, Sexual and Reproductive Health Research Center, Addiction Institute, School of Medicine, Mazandaran University of Medical Sciences, Sari, Iran; 4 Department of Psychiatry, School of Medicine, Mazandaran University of Medical Sciences, Sari, Iran; 5 Associate Professor of Epidemiology, Gastrointestinal Cancer Research Center, Non-Communicable Diseases Institute, Mazandaran University of Medical Sciences, Sari, Iran; 6 Obstetrics and Gynecology Surgeon, Department of Obstetrics and Gynecology, School of Medicine Mazandaran, University of Medical Sciences, Sari, Iran; 7 Department of Public Health and Community Medicine, School of Medicine, Tufts University, Tufts, USA; 8 Department of Biostatistics, Faculty of Paramedical Science, Shahid Beheshti University of Medical Sciences, Tehran, Iran; 9 Midwifery Counseling, Student Research Committee, Mazandaran University of Medical Sciences, Sari, Iran

**Keywords:** Psychology, Health care

## Abstract

Polycystic ovary syndrome is one of the commonest and complex endocrine disorders in females of reproductive age. Attention to self-care behaviors such as health-promoting behaviors can improve physiological and psychological conditions in women with PCOS. This study aimed to determine the efficacy of Social Network-Based Motivational Interviewing on health-promoting behaviors and quality of life of infertile women with polycystic ovary syndrome. In this randomized controlled trial, 60 infertile women with polycystic ovary syndrome were randomly selected and assigned to the interventio (n = 30) or a control group (n = 30). Participants in the control group received routine care. Those in the intervention group received five motivational group sessions in WhatsApp, which were conducted in five groups of six participants each. The data related to health-promoting behaviors and quality of life were collected through an online questionnaire. Both groups were followed up immediately and 2 months after the intervention. Data collected in the two stages were analyzed using the paired-samples t-test, Chi-square, and repeated measures analysis. No significant difference was observed between the two groups before the intervention means health-promoting behaviors and quality of life scores (p>0.05). Immediately and two months after the intervention, the mean scores of health-promoting behaviors and their domains increased in the intervention group. This increase is statistically compared to the first test, and the scores obtained by the control group in the second (p< 0.001, ES = 1.5) and the third test (p< 0.001, ES= 1.3) were significant. The score of the quality of life variable increase was not statistically significant compared to the first test and also to the scores obtained by the control group. However According to the Generalized estimating equations (GEE) test, the changes in quality of life score between the two groups in the three stages of measurement are statistically significant. WhatsApp support increases the health-promoting self-care behaviors rate and has favorable effects on follow-up.

*Trial registration*: Iranian Registry for Clinical Trial (the link to trial: https://www.irct.ir/trial/48089). Registered August 11 2020.

## Introduction

Polycystic ovary syndrome (PCOS) is one of the most common and complicated conditions that affect women's endocrine glands during their reproductive age^1^. According to Rotterdam criteria, its overall prevalence is 17/8 percent worldwide^2^ and 20 percent^3^ in Iran. According to Rotterdam consensus criteria in 2003, PCOS is defined based on the presence of at least two of the following three symptoms: oligo-ovulation, hyperandrogenism (clinical or biochemical) as well as the diagnosis of at least 12 or more than 12 small antral follicles on the ultrasound of ovaries^4^.

Women with this condition have a higher risk of infertility, endometrial cancer, type 2 diabetes, and cardiovascular diseases^3^. The rate of infertility among women with PCOS tends to be ten times higher than other women, which is generally associated with insulin resistance and hyperinsulinemia^5^. The prevalence of infertility has been reported in 13.35 percent of women with PCOS^6^. Nearly 90 to 95 percent of women who refer to infertility clinics due to anovulation are experiencing PCOS^7^. Suffering from PCOS not only lowers the quality of life and mental health significantly, but it also limits the women's ability to undertake physical and emotional challenges of everyday life. It also increases their physical pain, depletes their energy, reduces their sexual gratification, undermines their feminine identity and restricts their interpersonal interactions^2^. Among various factors that adversely affect these patients' quality of life in terms of physical aspects, one could refer to obesity, hirsutism, irregular menstrual cycles, and infertility. Also, lack of self-confidence, loss of concentration, and self-isolation account for the mental aspects of this condition^3^.

These symptoms could be alleviated by taking measures involving lifestyle, taking medication, or in some cases, by more invasive methods such as laparoscopic surgery^8^. Prior to medication intervention in the treatment of infertile women with PCOS, the importance of making changes to lifestyle, particularly weight loss, undertaking more physical exercise, giving up smoking, and reducing the consumption of alcoholic beverages, must be highlighted^9^.

Weight loss is being used to normalize androgens, treat insulin resistance, and establish ovulation as the core component and the first line of treatment in these patients^10,11^. 5–10 percent weight loss would bring about an improvement in clinical symptoms among the patients as well as the response to the treatment of infertility and the outcome of pregnancy^12^.

Evidence indicates that women with PCOS should improve their lifestyle synthetically to achieve a better result in their treatments. This is why serious attention to diet, physical activities, monitoring and management of behavior and emotions, accurate diagnosis of symptoms, and other kinds of self-care are essential for maintaining and boosting the quality of life in women with PCOS^13^.

One of the important ways of preparing patients for changing their lifestyle and assisting them with making tough decisions that arise in the course of treatment is psychological support and appropriate counseling methods such as behavioral therapy^14^, team psychological interventions^15^, and motivational interviews (MI)^16^. MI is based on Carl Rogers' client-centered approach to psychotherapy. It is a client-centered and counseling-oriented approach to boost intrinsic motivation for change by discovering, identifying, and resolving doubts and dualism^17^. The main objective of MI is to enhance clients' intrinsic motivation to the extent that willingness for change would originate from within the individual and not superficially imposed upon him from the outside. The principles of MI are as follows: expressing empathy, revealing the conflict between the current behavior and values and goals, forbearing clients' resistance, supporting self-efficiency, and avoiding arguments and disputes^18,19^. Unlike the standard methods of treatment, the aim of this approach is not to alleviate or eliminate the clinical symptoms, but rather it seeks to illustrate the tensions involving dangerous behaviors so that people can become aware of appropriate treatment strategies consistent with their values^20^.

Indicates that, in general, the patients who were exposed to motivational interviews were one and a half times more likely to improve in a broad spectrum of healthcare measures in comparison with the control groups^21,22^. Motivational interviews with motivation-enhancement mechanisms will increase self-care behaviors and promote health. Overall, a health-promoting lifestyle is a beneficial resource for boosting the quality of life, and it has a significant effect on the reduction of health-related expenses, increasing people's longevity, and improving the quality of their life^23^. Therefore, purposeful management focused on improving lifestyle with an emphasis on self-care is of great importance.

Considering that both the outcome and the target group determined in the leading study are not similar to any of the studies conducted outside and in Iran, and considering the contradictory results of the studies conducted in this group of patients in other outcomes and the importance of paying attention to the unbelieving segment of the society, this The study was designed as a randomized clinical trial to provide stronger evidence in support of the existing hypotheses on the effect of motivational interviewing on health-promoting behaviors and quality of life of infertile women with polycystic ovary syndrome. Also, since the issue of women's quality of life is a variable that is formed in the socio-cultural context and beliefs of any society; Therefore, this study paid attention to the reality of the traditional society of Iran, where the quality of life of women is influenced by many factors. It is hoped that the results of this study will help in improving the quality of life and improving the health of infertile women with PCOS.

The primary aim of this study was to determine the effectiveness of social network-based motivational interviews on health-promoting behaviors in infertile women with polycystic ovary syndrome immediately and two months after the intervention. Secondary objectives included: 1- Evaluating the effect of the motivational interview on quality of life 2- Acceptability 3- Feasibility.

## Methods

### Trial design and hypotheses

A randomized clinical trial was conducted to determine the effect of a WhatsApp social network-based motivational interviewing intervention in adopting Health-promoting behaviors and quality of life in infertile Iranian women with polycystic ovary syndrome.

Our central hypothesis was that the WhatsApp social network-based motivational interviewing intervention would significantly increase the health-promoting behaviors and quality of life in the intervention group from pre-test to post-test compared to the control group.

The study participants were assessed at baseline and 2-month follow-up. The Mazandaran University of Medical Sciences Research Ethics Board approved the study. The trial was registered on 11/08/2020, with the Iranian Registry of Clinical Trials with Registration number; Trial Id: 48,089 (identifier: IRCT20160619028528N4). Please see the Iranian Registry of Clinical Trials at https://www.irct.ir/trial/48089.

### Participants and setting

In summary, we conducted a pragmatic parallel-group randomized controlled trial in Sari, Iran, in 2020, in which 60 infertile women with PCOS. These women entered the study following receiving verbal consent via telephone and signing the online written consent. Inclusion criteria include infertile women with PCOS based on Rotterdam criteria according to the diagnosis of an obstetrics and gynecology surgeon with infertility treatment expertise, Age [15–40] Iranian literate. Exclusion criteria include: Having suffered from other chronic diseases during the last 3 months and Being under the care of a psychologist or a psychiatrist at and taking psychiatric medication, any history of significant depression or major family disputes and grief (death of close relatives) in the week leading up to the beginning of the study.

The study site and data collection sites were the infertility clinic of Imam Khomeini Hospital in Sari, Iran.

### Intervention group: motivational interviewing program Active phase (month 0-1)

To train the intervention group, first, the participants were asked whether they were active members of WhatsApp, and they were asked to install the mobile app. Afterward, a motivational interview was given to them in audio/video call and written forms, A deck of PowerPoint slides was also sent to them. Also, the participants were able to ask the researcher their questions in private. The motivational interview took place once a week. The training program was formulated according to the protocol produced by the research team. The contents of the sessions included the following: the first session was orientation; the second session was about emotions; the third session was about the positive and negative aspects of behavior and change, and the fourth and fifth sessions dealt with values and vision as well as the final assessment. (Table 1)

**Table 1:** Sessions description of study intervention.

Session one	Introduction group norms and processes, Understanding the anatomy and physiology of the reproductive system, Timeline commitment/Confidence level rating
Session two	clarify feelings topics related to ambivalence feeling the need to change behavior, the effect of emotional dimensions such as beliefs and low self-esteem
Session three	Brain storming practice, awareness of risks by weighing the costs and benefits the pros & cons exercise alternative states descriptor
Session four	Define values identify values the discrepancy between one's value and one's decision practice matching values and behavior
Session five	To reassess patient's commitment, confidence levels and motivation for change, summarize the exercises of the previous sessions in the form of presenting their visions and preparing to start the behavior change program

The intervention was conducted using the Camtasia 2019 software on WhatsApp. Each session lasted 60 minutes. The intervention was performed by a midwife trained in the area of the motivational interview. In the motivational interview, the following were used: balance of decision making, circle of preparation for change, increasing contrast between values and self-care behaviors, behavior control in different situations, and outlining a vision. In the self-care training program for improving the Health Behaviors and quality of life, the following concepts were discussed: raising awareness about PCOS, its side effects, teaching self-care behaviors including Lifestyle modification with an emphasis on behavioural and emotions management and dietary and exercise.

### Follow-up phase (month 1- 2)

At this stage, although motivational interview sessions were eliminated, participants still had access to a coach and trained resources in WhatsApp groups. Participants were encouraged to continue the behavior change process and ask their coaching if they had any questions.

### Control group

The control group received routine clinical treatments and counseling related to infertility and PCOS.They were invited to complete the questionnaire immediately and two months after the intervention. This ccontrol group also received motivational interviews at the end study of the with the same quality in five sessions.

### Measurement

A questionnaire was formulated by the research team in three parts, including personal information, family information, and information about fertility to examine the demographic and medical features of the patients in this study.

-Health promotion behavior through self-care measured using The Health-Promoting Lifestyle Profile II (HPLP-II). summation of 52-items behavior rating scale employs a four-point response format to measure the frequency of self-reported HPBs in the domains of health responsibility, physical activity, nutrition, spiritual growth, interpersonal relations and stress management. There is a four-point Likert type scale for each item, ranging from 1 (“never”) to 4 (“routinely”). Higher scores indicate greater participation in health-promoting behaviors^24^. The Persian version HPLP-II, which was used in this study is an instrument that has been acceptable content validated by Mohammadi Zeidi et al (Cronbach's alpha 0/82)^25^.

Quality of Life measured using the modified PCOS health-related quality of life questionnaire (MPCOSQ). The MPCOSQ include 30 questions from six domains: emotional disturbances (8 items), hirsutism (5 items), infertility (4 items), weight (5 items), menstrual (4 items) and acne (4 items). Each item is associated with a seven-point Likert scale, in which a score of 7 suggest no problems or difficulties and 1 indicates maximum impairment on that item^26^. Psychometric properties of MPCOSQ in Iranian population have been verified by Bazarganipour et al (Cronbach's alpha ranging from (0.76 to 0.92)^27^.

Acceptability was post-intervention assessed with the Client Satisfaction Questionnaire-8. It encompasses 8 questions, each having 4 responses. The responses take a score between 1-4 based on their degree of positivity or negativity (4-very positive, 3-positive, 2-negative, 1-very negative). The minimum and maximum scores of the subject on this scale are 8 and32respectively. Higher scores indicate greater treatment satisfaction.this questionnaire has been reported to be benefits good construct and content validity .This questionnaire was translated into Persian by Shareh its content validity was confirmed by clinical psychologists and psychiatrists and its reliability was estimated^28–30^. Measures of feasibility of conducting this study was included study enrollment and intervention completion.

### Randomization, allocation, and blinding

Sampling in this study were be available in the first stage in order to identify eligible women with polycystic ovary syndrome. In the next stage, 60 eligible patients were be assigned to an intervention group (motivational interview recipient) and a control group (routine care) using computer-generated random allocation sequence be the randomized blocking (block size 4 and 6) method. Allocation was concealed in sequentially numbered opaque envelopes. To prevent observer bias, a single observer (the researcher) conducted all sampling steps. Random allocation was utilized to prevent selection bias and conceal the allocation of subjects. Additionally, statistical intention-to-treat (ITT) analysis and blinded assessors were employed to control for detection bias. While blinding participants and instructors was not feasible in this study. During the follow-up phase, one individual from the control group was excluded due to withdrawal, and another from the control group was excluded due to non-response.

### Sample size

According to a search of available databases, no studies were found that examined the effect of motivational interviewing on infertile women with PCOS. For this purpose, the sample size of 60 people (control and intervention group of 30 people each) was estimated for selection.At the end of the study, the test power was determined to reject the null hypothesis.

### Statistical analysis

We analyzed data based on an intent-to-treat approach and thus, included all participants with at least valid baseline data according to group allocation.

SPSS version 26.0(IBM Corporation,New York,USA) was used to analyze the data. Descriptive statistics such as frequency (N) and percentage (%), mean (M), and Standard Deviation (SD) were used to summarize the demographic data of the participants. Chi-square test and independent t-test were used to compare baseline and demographic data. The relative frequency of each case was obtained. P < 0.05 was considered significant.

Repeated Measures test and Generalized Estimating Equations test was used to compare the health-promoting behaviors scores and quality of life before, immediately, and after the intervention in each group. In addition to evaluating the effect of the intervention, the effect size(EF) and Number Needed to Treat (NNT) were compared with other interventions. (Table 4).

### Ethics approval and consent to participate

Ethical approval for the study was granted by the ethics committee of Mazandaran University of Medical Sciences (IR.MAZUMS.REC.1399.191). Online informed consent was obtained from each participant before data collection. All the methods were performed in accordance with relevant guidelines and regulations 31 codes of ethics in Iranian biomedical research and Declaration of Helsinki.

## Results

### Study flow and participant retention in the trial

**Figure 1:**
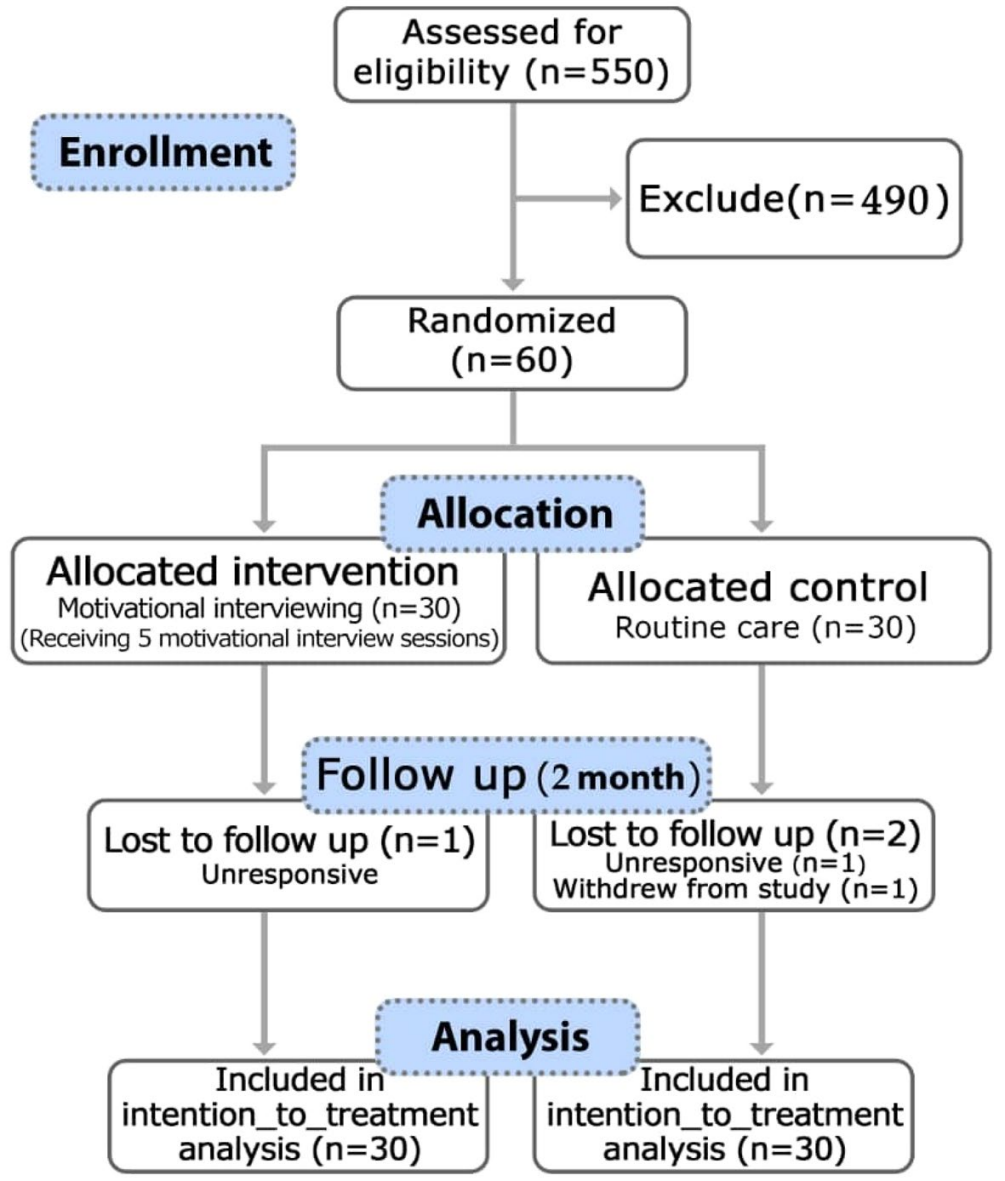
Follow-up of study participants.

The study occurred between August 2020 and February 2021. In total, 550 individuals expressed underwent assessment for eligibility. 490 had not eligibility criteria, and a total of 60 were randomly allocated to either intervention (n = 30) or comparator (n = 30). Figure 1 outlines the study flow to 2 months, corresponding to the time point of the primary outcome.

In the follow-up phase, one person from the control group was excluded from the study due to withdrawal, and one person from the control group was excluded from the study due to non-response. In the second stage of follow-up, one person from the control group was excluded from the study due to non-response (Fig 1). In summary, trial retention was 94% at 2 months (i.e., 96% of participants in the intervention group and 93% of participants in the wait-list control group remained in the study).

### Baseline characteristics

As it was expected, due to randomization, the groups initially had balance in terms of social-demographic characteristics and fertility history.

The average age of women was 30.97 in the intervention group and 28.67 in the control group. The average age of menarche in both intervention and control groups was around 13. 60 percent of women in the intervention group and 66 percent of them in the control group had primary infertility, and 38 percent of women in the intervention group and 32 percent of them in the control group had secondary infertility. Around 60 percent of women in the intervention group and 43 percent of them in the control group had Phenotype-C of PCOS. The two groups were homogeneous in terms of statistics in of the variables (age, education, job, income, age of menarche, menstrual intervals, Phenotype, Reproductive history, Medication and Other treatments) (Table 2).

**Table 2:** Baseline Participant Characteristics: Socio-demographics and fertility variables.

Characteristic	Intervention group (n = 30)	Control group (n = 30)	p-value
Age, mean (SD)	30.97 (4.18)	28.67(5.30)	0.067
Education, N. (%)			
High school or less	20 (66.7)	26 (86.7)	0.261
Greater than high school	10 (33.3)	4 (12.7)	
Husband Education, N. (%)			
High school or less	16 (53.3)	17 (56.7)	0.296
Greater than high school	14 (46.7)	13 (43.3)	
Job, N. (%)			
Employee	7 (23.3)	5 (16.7)	0.232
Unemployed	23 (76.7)	25 (83.3	
Husband Job, N. (%)			
Employee	29 (87.16)	28 (93.4)	0.499
Unemployed	1 (3.3)	2 (6.7)	
Income, N. (%)			
Enough	23 (76.7)	19 (63.3)	0.756
Not enough	7 (23.3)	11 (36.7)	
Age of menarche ,mean (SD)	13.57 (1.50)	13.20 (3.13)	0.566
Menstrual intervals, N. (%)			
Poly menorrhea	2 (6.7)	1(3.3)	0.973
Oligo menorrhea	6 (20)	7 (23.3)	
Amenorrhea	1 (3.3)	1 (3.3)	
Regular	11 (36.7)	10 (33.3)	
Irregular	10 (33.3)	11(36.7)	
Reproductive history, N. (%)			
Primary infertility	18 (60)	20 (66.7)	0.839
Secondary infertility	12 (40)	10 (33.3)	
Medication, N. (%)			
Yes	10 (33.3)	7 (23.3)	0.482
No	20 (66.7)	23 (76.7)	
Other treatments, N. (%)			
Yes	3 (10)	6 (20)	0.204
No	27 (90)	24 (80)	
Phenotype PCOS, N. (%)			
A	7 (23.3)	7 (23.3)	0.480
B	2 (6.7)	4 (12.7)	
C	18 (60)	13 (43.3)	
D	3 (10)	6(20)	

*SD* standard deviation.

### Primary outcome

Compared to the control group, the intervention group increased their health-promoting behaviors more following the program for the motivational interview. This difference in the increase in the intervention group was observed immediately and two months following the intervention. Self-promoting behaviors immediately and two months after the intervention in both of the groups, the recurring measurements indicate a significant difference in terms of statistics (P=0/000) (Table3). The Power of study was calculated to be 99% concerning the primary outcome.

**Table 3:** Comparison of the HPLP II and MPCOSQ variables before and after the intervention in the intervention and the control group.

Variables	Baseline			1 month			2 month		
	Control group	Intervention group	p-value*	Control group	Intervention group	p-value*	Control group	Intervention group	p-value*
	Mean (SD)	Mean (SD)		Mean (SD)	Mean (SD)		Mean (SD)	Mean (SD)	
HPLP II	1 14.63 (14.52)	117.80 (20.06)	0.487	115.80 (18.69)	144.40 (19.15)	0.001	116.76 (17.03)	141.50 (20.41)	0.001
Nutrition	20.86 (4.53)	21.16 (4.51)	0.798	23.80 (4.93)	25.43 (4.07)	0.168	23.00 (4. 22)	25.03 (4. 06)	0.062
Physical activity	14.33 (3.04)	14.60 (3.59)	0.758	14.07 (3.79)	19.13 (3.26)	0.000	14.83 (3. 80)	19.03 (3. 48)	0.000
Health responsibility	20.00 (2.76)	20.00 (4.51)	1	18.66 (4.58)	23.73 (4.29)	0.001	18.76 (3. 74)	22.86 (5. 17)	0.000
Stress management	17.06 (3.30)	18.06 (3.72)	0.275	17.86 (4.09)	22.50 (3.75)	0.000	17.56 (4. 04)	22.526 (3. 71)	0.000
Interpersonal relations	21.50 (3.25)	22.56 (5.02)	0.334	21.50 (4.30)	26.26 (4. 40)	0.000	22.06 (3. 41)	25.93 (4. 73)	0.001
Spiritual growth	20.86 (3.79)	2 1.40 (4.91)	0.640	20.73 (6.06)	27.20 (5.45)	0.000	20.60 (4. 92)	26.53 (5. 71)	0.000
MPCOSQ	122.03 (33.54)	115.33 (36.11)	0. 615	126.66 (32.53)	136.73 (29.92)	0.128	122.43 (36.14)	133.70 (31.77)	0.228

HPLP II Health-Promoting Lifestyle Profile II, MPCOSQ: The modified polycystic ovary syndrome health-related quality-of-life questionnaire, *SD* standard deviation.

*Result of the Independent-Sample T Test.

p ≤ 0.05 significant.

### Secondary outcomes

#### Quality of life

The mean of the quality of life immediately and two months after intervention has increased in the two intervention groups; however, it has not been statistically significant. (Table 3)

According to the GEE test, the changes in quality of life score between the two groups in the three stages of measurement are statistically significant. (Table 4).

**Table 4:** Comparison EF and NNT of the HPLP II and MPCOSQ variables after the intervention.

Variables	1 month		2 month		p-value
	ES(95%CI)	NNT	ES(95%CI)	NNT	
HPLP II	1.51 (0.938–2.085 )	1.4	1.31 (0.758–1.874)	1.5	0.001*
MPCOSQ	0.32 (− 0.187–0.832)	5.5	0.33 (0.178–0.841)	5.4	0.001**

*EF* Effect Size, *NNT* Number Needed to Treat.

*Result of the Analysis of Repeated measures analysis.

**Result of the Analysis of generalized estimating equations (GEE).

### Acceptability

CSQ-8 results suggested high levels of satisfaction with the program. According to CSQ-8 results, the mean total score in the control group who received routine care was less satisfactory. While the WhatsApp-based motivational interviewing program met achieved higher satisfaction (Table 5).

**Table 5:** The Client Satisfaction Questionnaire (CSQ-8).

Questions	Mean (SD)		p-value
	Control Group	Intervention Group	
Total Mean Score	19. 90 (6.02)	25.90 (1.84)	0.001
1. Quality of service?	2.53 (0.57)	3.23 (0.62)	0.001
2. Kind of service you wanted?	2.57 (0.93)	3.23 (0.43)	0.001
3. Extent program met your needs?	1.93 (0.64)	3.17 (0.59)	0.001
4. Recommend program to friend?	2.63 (0.99)	3.53 (0.50)	0.001
5. Satisfaction with the amount of help received?	2.37 (0.89)	3.07 (0.25)	0.001
6. Services helped you to deal with problems?	2.77 (0.62)	3.10 (0.40)	0.017
7. Overall satisfaction with the service?	2.50 (0.97)	3.27 (0.52)	0.001
8. Return to program for help?	2.60 (0.96)	3.30 (0.53)	0.001

Responses for individual items range from 1–4 (median score is 2.5), with higher scores indicating higher satisfaction; total scores can range from 8–32 (median score is 20).

### Feasibility

As part of the feasibility study, the participants' Adherence was determined as a percentage. The table above shows that more than 97% of the participants attended all the sessions.

When considering all individuals who were allocated to the intervention group (n = 30), compliance to the in-person component of the intervention was as follows: 96.66% attended session one; 100% attended session two; 100% attended session three; 93.33% attended session four; and attended sessions five 96.66 %.

## Discussion

This study was a randomized controlled clinical trial of a pragmatic type with aimed to determine the effectiveness of social network-based motivational interviews on health-promoting behaviors and quality of life in infertile women with polycystic ovary syndrome.

The findings of this study showed that health-promoting behaviors among the participants in the intervention group significantly increased after the WhatsApp social network-based motivational interviewing intervention compared with the control group. This finding is in line with similar studies, which showed that mobile phone-based and WhatsApp interventions could be an effective strategy to improve the behavior change process^31,32^. Furthermore, analyses showed that these individuals maintained these outcomes 2 months later (within-group changes), following a minimally-supported phase that involved only online technical supports.

With a substantial proportion of women in Iran and the world living with one or more modifiable risk factors for polycystic ovary syndrome and considering the rise in the costs associated with health care, lifestyle programs are essential to prevent and assist with the treatment and management of disease burden. Psychological interventions have been carried out to improve health-promoting behaviors and quality of life in other chronic diseases, the majority of which have indicated that they result in the improvement or reduction in the symptoms of chronic diseases. By improving adherence to hygienic and self-care behaviors, the motivational interview could significantly positively impact self-promoting behaviors among patients suffering from heart disease and depression. It is likely to be beneficial for body mass index in patients with type 2 diabetes^33,34^. It has been indicated that the intervention of mindfulness-based programs for infertility (MBPI) has promoted self-efficiency in women with infertility^35^. Cognitive-behavioral Group Therapy (CBGT) and lifestyle intervention group in infertile women could boost their quality of life and health indices^36,37^.

The evidence from the Motivational interviewing integrated with social network program supports positive behavior changes. Despite positive changes, the program did not lead to significant changes in quality of life . We speculate that the components of our program and length of follow-up might not have been intense enough to elicit significant changes in these outcomes^38–40^.

The current article provides an effect size estimate and number needed to treat. Estimating the size of the effect provides more information for judging the practical significance. This leads to clinical decisions being based on scientific evidence studies. The effect size of health-promoting behaviors was immediately and two months after the intervention with large effect size.

The NNT number was calculated for health-promoting behaviors immediately after the intervention (1.4) and two months after the intervention (1.5) was calculated. in other words, approximately one in two infertile women in the treated group, immediately and two months after the intervention, one show improvement in health-promoting behaviors. Previously, a study by Cleary and colleagues of patients with mental illness aimed to evaluate the effectiveness of psychological interventions in reducing substance use by people with a mental illness. The number needed to treat the motivational interview was reported in two studies (NNT= 2 and NNT= 4), respectively^41^. Another study was comparing pharmacological and psychological interventions in the treatment of depression after stroke reported an (NNT= 7) for motivational interview intervention^42^. By comparing the present study with the studies mentioned earlier, it could be concluded that this study has presented effective results in terms of clinical practicality.

Using the principles of motivational interviewing in patients provides the possibility of changing behavior that contributes to positive health outcomes. These changes can have an important impact on the management of major diseases of infertile women. It is appropriate to include the principles of motivational interviewing during all kinds of encounters between infertility clinic treatment staff and patients. Although this behavior change can be established in short sessions, follow-up is useful and often necessary to help achieve long-term results. Incorporating motivational interviewing techniques into daily clinical encounters increases the ability of obstetrics and gynecology teams in infertility clinics to serve patients.

Counseling in infertility is different from other counseling in other diseases. Counselors are often faced with the psychological suffering of an unfulfilled wish or goal from a third person (child) who does not yet exist in all the main areas of counseling. The ability to cope with the psychological and emotional effects of this process should be considered in the course of treatment.

Based on the protocol designed to carry out the intervention in the current study and according to the results, it can be said that by accompanying and counseling, giving information and knowing the factors affecting polycystic ovary syndrome in affected infertile women, increasing self-care, The treatment steps of assisted reproductive methods, the effect of lifestyle on infertility and PCOS, the importance of weight loss and nutritional management in improving the outcome of treatment, communication skills training, stress coping skills, helping to remember information, helping to increase the sense of participation, decision Better acceptance and active participation of the patient in the stages of infertility treatment and help in self-management of PCOS symptoms will ultimately increase self-care and improve the quality of life of infertile women with PCOS. Therefore, the results of this study and the content of the intervention protocol can be helpful in infertility centers as a complementary method.

One of the motivations of conducting research is to use its results to improve the quality of care and services to clients. The specialized midwifery team should be able to help improve the current situation by applying new knowledge and skills in the field of health promotion and paying attention to psychological aspects in infertile women with polycystic ovary syndrome.

Finally, integration of remote delivery of motivational interviewing appears feasible and may even provide participants with options that appeal to their needs. Further study is required to improve retention and adherence using pragmatic methods.

### Limitations and strengths

The limitations of this study were the short follow-up period. Also, in the study, data were obtained based on a self-report questionnaire, which might cause potential bias in the results. In online counseling, the inexistence of non-verbal signals or body language increases the possibility of an inappropriate relationship between the patient and the therapist. On the other hand, people vary in terms of their ability to express their thoughts and feelings in written words. The present study attempted to prevent this limitation as much as possible by making video calls and sending voice messages. Some of the other limitations of online counseling which the participants occasionally face are the breakdown of equipment and online services.

One of the strengths of this study is that according to the research team, this study is the first interventional study of infertility women with polycystic ovary syndrome by remote delivery of motivational interviewing.

Also, in the present study, the structure of the intervention was established by presenting a protocol developed by the expert team. According to sample size, a procedure for sampling, and the participation rate, these study’s results are broadly ae and different types of infertility centers and situations.

### Future directions

Further study is required to improve retention and adherence in health-promoting behaviors and quality of life using pragmatic methods. It should be mentioned here that counseling should be taken along with medical methods on health- promoting behaviors and quality of Life among Infertile women with Polycystic Ovary Syndrome.

## Conclusions

This study results suggest that these individuals can maintain these behaviors 2 months later with minimal support, showing promise for long-term sustainability. Process evaluation data from a clinical trial of the WhatsApp-based motivational interviewing Program demonstrated that the intervention was feasible and acceptable to participants.

## Data Availability

The datasets used and/or analyzed during the current study are available from the corresponding author on request.
